# *Salmonella enterica* Optimizes Metabolism After Addition of Acyl-Homoserine Lactone Under Anaerobic Conditions

**DOI:** 10.3389/fmicb.2020.01459

**Published:** 2020-07-28

**Authors:** Deisy G. Carneiro, Felipe A. Almeida, Ananda P. Aguilar, Nívea M. Vieira, Uelinton M. Pinto, Tiago A. O. Mendes, Maria Cristina D. Vanetti

**Affiliations:** ^1^Department of Microbiology, Universidade Federal de Viçosa, Viçosa, Brazil; ^2^Department of Nutrition, Universidade Federal de Juiz de Fora, Governador Valadares, Brazil; ^3^Department of Biochemistry and Molecular Biology, Universidade Federal de Viçosa, Viçosa, Brazil; ^4^Department of Food and Experimental Nutrition, Food Research Center, Universidade de São Paulo, São Paulo, Brazil

**Keywords:** quorum sensing, autoinducer, growth stage, glucose uptake, metabolic pathway

## Abstract

Acyl-homoserine lactones (AHLs) are quorum sensing (QS) signaling molecules that mediate cell-to-cell communication in Gram-negative bacteria. *Salmonella* does not produce AHL, however, it can recognize AHLs produced by other species through SdiA protein modulating important cellular functions. In this work, the influence of the *N*-dodecanoyl-DL-homoserine lactone (C12-HSL) on glucose consumption, metabolic profile, and gene expression of *Salmonella* throughout the cultivation time in Tryptic Soy Broth (TSB) under anaerobic conditions was evaluated. Analysis of the supernatant culture in high-performance liquid chromatography (HPLC) revealed lower glucose uptake after 4 and 6 h of the addition of C12-HSL. Gas chromatography-mass spectrometry (GC-MS) based analysis of the intracellular metabolites revealed C12-HSL perturbation in the abundance levels of metabolites related to the metabolic pathways of glycerolipids, purines, amino acids, and aminoacyl-tRNA biosynthesis. The real-time quantitative PCR (RT-qPCR) indicated that *Salmonella* increase expression of genes associated with nucleoside degradation and quantification of metabolites supported the induction of pentose phosphate pathway to ensure growth under lower glucose consumption. The obtained data suggest an important role of C12-HSL in the optimization of metabolism at a situation of high population densities.

## Introduction

*Salmonella* is a facultative intracellular enteric pathogen that infects both humans and animals (Andino and Hanning, 2015). Gastrointestinal diseases of infectious origin caused by the ingestion of food contaminated by this pathogen constitute a significant public health problem in the world (Santhi et al., 2012). It is estimated that 535,000 cases of invasive non-typhoid *Salmonella* disease occurred in 2017, causing 77,500 deaths (Stanaway et al., 2019). Among the serotypes involved, *Salmonella* Enteritidis is one of the most commonly associated (Balasubramanian et al., 2019).

*Salmonella* is a robust pathogen and has a wide repertoire of strategies to cause infection in the host. These include genes that encode virulence factors, usually grouped in pathogenicity islands (Fookes et al., 2011; Kaur and Jain, 2012; Nieto et al., 2016), as well as, the presence of virulence plasmid *p*SLT, fimbriae, flagellum, in addition to the capacity to resist the stresses found in the host (Fabrega and Vila, 2013; Runkel et al., 2013). Besides, the ability to adjust the metabolism to adapt to the physical conditions and available nutrients found in the host during infection seems to be fundamental for the success of *Salmonella* as a pathogen (Dandekar et al., 2015; Rivera-Chávez and Andreas, 2015; Herrero-Fresno and Olsen, 2018). In addition, the regulation of these phenotypes can occur in a synchronized way, through a cellular communication mechanism called quorum sensing (QS).

The QS is a mechanism used by many bacteria that is based on the production of signaling molecules called auto-inducers (AIs). Usually, AIs diffuse or are transported to the environment where they accumulate and, upon reaching a threshold concentration, they bind to intracellular or extracellular receptors regulating the expression of target genes (Fuqua et al., 1994; Keller and Surette, 2006; Hense and Schuster, 2015). In *Salmonella*, three QS systems have already been described and are mediated by AI-1, AI-2, and AI-3 (Walters and Sperandio, 2006; Simões et al., 2010).

In Gram-negative bacteria, the most studied mechanism of QS is mediated by acyl-homoserine lactone (AHL), an AI-1, synthesized by LuxI protein homologs (Papenfort and Bassler, 2016). *Salmonella* does not contain the *luxI* gene in its genome; thus, it does not synthesize AI-1. However, *Salmonella* can detect and respond to AHLs produced by other species because it encodes a transcriptional regulator homologous to LuxR proteins called SdiA (Michael et al., 2001; Banerjee and Ray, 2016).

Knowing phenotypes regulated by the AI-1 mediated QS system in *Salmonella* is of interest and could help the comprehension of the pathogen’s virulence. For instance, in *Salmonella* Typhimurium, SdiA positively regulates the expression of the *rck* (resistance to complement killing) operon, which codes for *pefI*, *srgD*, *srgA*, *srgB*, *rck*, and *srgC* genes, and it is found in the virulence plasmid *p*SLT (Ahmer et al., 1998; Soares and Ahmer, 2011; Sabag-Daigle et al., 2012). Also, the addition of 50 nmol L^–1^ of *N*-dodecanoyl homoserine lactone (C12-HSL) promoted an increase in the expression of the virulence genes and those involved in biofilm formation in *Salmonella* Enteritidis PT4 578 (Campos-Galvão et al., 2016). Moreover, the biofilm formed after the addition of C12-HSL is more compact and mature when compared to other AHLs with a lower carbon chain (Campos-Galvão et al., 2016). This could be explained by its higher binding affinity to the SdiA protein when compared to other AHLs through molecular docking studies (Almeida et al., 2016). Besides C12-HSL, other AHLs tested did not influence the growth of *Salmonella* Enteritidis PT4 578 cultivated in anaerobic Tryptic Soy Broth (TSB) medium at 37°C (Campos-Galvão et al., 2015; Almeida et al., 2017b). Cultivation of *Salmonella* Enteritidis PT4 578 in anaerobic TSB medium supplemented with C12-HSL also led to alterations in protein abundance and levels of organic acids associated with the early stationary phase of growth (Almeida et al., 2017a). It also increases the expression of thiol-related proteins and thiol, as well as the alteration in the fatty acid profile (Almeida et al., 2018). However, despite the evidence of the association between cell density-dependent gene expression, so far, there is a paucity of information related to the overall impact of AI-1 on the metabolism of *Salmonella* Enteritidis.

In this work, high-performance liquid chromatography (HPLC), metabolomics based gas chromatography-mass spectrometry (GC-MS) and real-time quantitative PCR (RT-qPCR) were used to quantitatively investigate the metabolic response of *Salmonella* Enteritidis PT4 578 to the addition of C12-HSL in TSB under anaerobic conditions.

## Materials and Methods

### Bacterial Strain

The *Salmonella enterica* serovar Enteritidis PT4 578 (GenBank: 16S ribosomal RNA gene MF066708.1) used in this study was isolated from chicken meat and donated by the Oswaldo Cruz Foundation (FIOCRUZ) Rio de Janeiro, Brazil. The culture was maintained in Luria Bertani broth (LB) (tryptone 1%, yeast extract 0.5% and NaCl 0.4%) with 20% (vol/vol) sterile glycerol at −20°C. All experiments were performed in laboratories with biosafety level 2.

### Culture Medium

Cell culture was performed in Tryptic Soy Broth (TSB; Sigma-Aldrich, St. Louis, MO, United States) prepared with CO_2_ under O_2_ free conditions, dispensed in anaerobic flasks sealed with a butyl rubber stopper and autoclaved at 121°C for 15 min.

### Culture Conditions

Before each experiment, cells were cultured two consecutive times in anaerobic bottles containing 10 mL of anaerobic TSB and incubated for 24 h at 37°C. Then, 0.2 mL were transferred to 20 mL of anaerobic TSB and incubated for 4 h at 37°C. Cells were centrifuged at 12,000 *g* for 10 min at 4°C (Sorvall, Waltham, MA, United States), and washed twice with 1 mL of saline 0.85% (wt/vol) and, finally, resuspended in 5 mL of saline 0.85%. The optical density of the cells was standardized at 0.1 at 600 nm (OD_600nm_) using a spectrophotometer (Thermo Fisher Scientific, Vantaa, Uusimaa, Finland) (Almeida et al., 2017b), which corresponds to, approximately, 10^7^ CFU.mL^–1^. The *N*-dodecanoyl-DL-homoserine lactone (C12-HSL; PubChem CID:11565426; Fluka, St. Gallen, Buchs, Switzerland) was suspended in acetonitrile (Merck, Darmstadt, Hessen, Germany) at a concentration of 10 mmol.L^–1^. Three flasks containing 90 mL of anaerobic TSB were supplemented with 500 μL of C12-HSL to a final concentration of 50 nmol.L^–1^, and the other three flasks were supplemented with the same volume of acetonitrile as a control. The choice of C12-HSL was based on studies showing its increased effect on biofilm formation by *Salmonella* compared to other AHLs with shorter carbon chains due to its higher affinity to SdiA. The concentration too was based on those same studies (Campos-Galvão et al., 2015, 2016; Almeida et al., 2016, 2017b, 2018). The acetonitrile concentration was less than 1% (vol/vol) in the culture medium since according to Michael et al. (2001), this concentration does not exhibit an effect on the growth and response of *Salmonella* to AHL. A total of 90 mL of medium was inoculated with an aliquot of 10 mL of the standardized inoculum resulting in a final volume of 100 mL and incubated at 37°C for 36 h. Samples were taken at specific time points of 4, 6, 7, 12, 24, and 36 h.

### Determination of Growth, pH, and Titratable Acidity

*Salmonella* growth was done by the drop plate method on Plate Count Agar (PCA, Himedia, Mumbai, Maharashtra, India) (Morton, 2001). The plates were incubated at 37°C for 36 h. Colony counts were manually performed and expressed as log CFU.mL^–1^. A portion of 2.5 mL of culture was also withdrawn and the pH was measured in potentiometer (Hanna Instruments, Woonsocket, RI, United States) and the titration was performed with 0.1 N sodium hydroxide (NaOH) in the presence of 1% (wt/vol) phenolphthalein. The results of titratable acidity were expressed as eq.L^–1^ of acids obtained.

### Analysis of Glucose Consumption

Glucose consumption was assessed by the quantification of glucose present in the extracellular medium by high-performance liquid chromatography (HPLC). At pre-established times, including initial growth time of 0 h, 2 mL of the culture were removed, immediately frozen in liquid nitrogen, and stored at −80°C until analysis. The sample was centrifuged at 12,000 *g* for 10 min, filtered on cellulose nitrate membrane and 1 mL of each sample was analyzed in HPLC Dionex Ultimate 3000 (Dionex Corporation, Sunnyvale, CA, United States) coupled to a refractive index Shodex RI-101 (Dionex Corporation, Sunnyvale, CA, United States), maintained at 40°C. Aminex^®^ HPX-87H ion-exchange column 4.6 mm × 300 nm 0.45 μm (Bio-Rad, Hercules, CA, United States), maintained at 45°C and a Micro-Guard Cation H column (Bio-Rad, Hercules, CA, United States) were used (Bento et al., 2015). The mobile phase contained 5 mmol.L^–1^ sulfuric acid (H_2_SO_4_) (Sigma-Aldrich, St. Louis, MO, United States), the flow was 0.7 mL.min^–1^, and the injection of 20 μL. The HPLC was calibrated with the standard glucose curve, prepared at a final concentration of 25 mmol.L^–1^.

### Analysis of Intracellular Metabolites

#### Sampling and Quenching of Cell Metabolism

A total of 10 mL was harvested at each time of sampling and immediately added into 15 mL of 60% methanol/water solution at −6°C for quenching of cellular metabolism, frozen in liquid nitrogen, and stored at −80°C until extraction (Winder et al., 2008).

#### Intracellular Metabolite Extraction

The samples were centrifuged at 12,000 *g* for 10 min at 4°C and intracellular metabolites were extracted from the quenched cell pellet using 1.5 mL of a mixture containing water, methanol (Sigma-Aldrich, St. Louis, MO, United States) and chloroform (Sigma-Aldrich, St. Louis, MO, United States) at −6°C in the proportions 1:2.5:1 following the protocol described by Vital et al. (2017). In each sample, 60 μL of ribitol (0.2 mg.mL^–1^ stock in ultrapure water) (Sigma-Aldrich, St. Louis, MO, United States) were added as an internal quantitative standard. The samples were agitated in thermomixer (Eppendorf, Hamburg, Hamburg, Germany) at 950 rpm for 10 min at 4°C. Subsequently, the samples were centrifuged at 11,000 *g* for 10 min at 4°C, and 1 mL of the supernatant was transferred to a new microtube containing 750 μL of ultrapure water. The samples were homogenized in a vortex and centrifuged at 14,000 *g* for 15 min at 4°C. The upper phase (polar phase) was collected and fractionated into aliquots in 1.5 mL microtubes. Aliquots of 200 μL were evaporated in a centrifugal vacuum concentrator (Speedvac; Eppendorf, Hamburg, Hamburg, Germany). The samples were stored at −80°C until derivatization.

#### Chemical Derivatization

The samples were derivatized according to the protocol described by Lisec et al. (2006). In summary, 40 μL of methoxyamine hydrochloride (Sigma-Aldrich, St. Louis, MO, United States) at 20 mg.mL^–1^ in pure pyridine (Merck, Darmstadt, Hessen, Germany) were added to each tube. The samples were shaken in thermomixer at 950 rpm for 2 h at 37°C. A volume of 70 μL of a solution of N-methyl-N-(trimethylsilyl) trifluoroacetamide (MSTFA; Sigma-Aldrich, St. Louis, MO, United States) with 40 μL.mL^–1^ of a mix of fatty acid methyl esters (FAMEs; Sigma-Aldrich, St. Louis, MO, United States) was added with standards for the retention time. The samples were shaken in thermomixer at 950 rpm for 30 min at 37°C. Finally, 90 μL of each sample were transferred to glass vials suitable for GC-MS analysis.

#### Gas Chromatography-Mass Spectrometry (GC-MS) Analysis

The samples were analyzed using a system of gas chromatography time-of-flight mass spectrometry (GC-TOF-MS), chromatograph Agilent Technologies 7890A spectrometer Leco, TruTOF^®^ HT TOFMS. The column used was a DB-35ms 30 m × 0.32 mm, 0.25 μm capillary column (Agilent Technologies, Santa Clara, CA, United States). The parameters of injection and analysis were defined according to Lisec et al. (2006). An aliquot of 1 μL of each sample was injected in splitless mode with the injector temperature at 230°C. The flow of helium gas through the capillary column was adjusted to 2 mL.min^–1^. The temperature remained isothermal for 2 min at 80°C then, increased by 15°C per min up to 330°C and, that temperature was maintained for 6 min.

#### Data Mining, Data Normalization

The chromatograms had their baseline corrected, and the compilation deconvolution algorithm provided by the software ChromaTOF (LECO, Mönchengladbach, Nordrhein-Westfalen, Germany) was used. The deconvoluted spectra were used to assign the peaks using TagSearch software (Cuadros-Inostroza et al., 2009), and spectral mass libraries of compounds derived from trimethyl silicon (TMS) obtained from the Max Planck Institute for Plant Molecular Physiology^1^. Areas of peak chromatography were verified and normalized by the peak area corresponding to the internal ribitol standard. The raw and normalized data were submitted to repository Metabolomics Workbench^2^ under track ID 1977.

#### Real-Time Quantitative PCR (RT-qPCR)

An RT-qPCR analysis was conducted to check the gene expression of *sdiA* and selected genes of purine metabolism *deoD*, *deoR*, *add*, and *hpt*. The culture of *Salmonella* Enteritidis PT4 was obtained as described in item 2.3. The total RNA was extracted in triplicate with Trizol^®^ Reagent (Invitrogen, Carlsbad, CA, United States) according to the supplier’s instructions. The quality of the RNA was evaluated in agarose gel, and the amount was estimated by measuring the absorbance at 260 nm in Nanodrop (Thermo Fisher Scientific, Waltham, MA, United States). A total of 1 μg was treated with RQ1 DNase I- RNase free (Promega, Madison, WI, United States). Complementary DNA (cDNA) synthesis was performed using 1 μg treated RNA and the ImProm-II Reverse Transcription kit with Random Hexamer Primer according to the manufacturer’s instructions. The program GenScript was used to draw the specifics primers for the gene encoding the SdiA protein and some genes of the purine metabolism *deoD*, *deoR*, *add*, and *hpt* (Table 1) using the *S. enterica* subsp. *enterica* serovar Enteritidis strain P125109 (GenBank: NC_011294.1) genome as a template. The self-annealing loops and dimers formation was evaluated using the Oligo Explorer tool and the primer specificity was verified by electronic PCR. The expressions of endogenous gene 16S (Johny et al., 2017) were used to normalize the data. RT-qPCR was performed with Sybr Green I Master Mix (Promega, Madison, WI, United States) in the 96-well plates in a Bio-Rad C1000 Thermal Cycler under custom thermal cycling conditions (denaturation step at 95°C for 2 min, followed by 40 cycles of a denaturation step at 95°C for 15 s, and an annealing/elongation step at 60°C for 1 min). The efficiency of amplification was determined by running a standard curve for each primer with serial dilutions DNA and calculated with the formula *E* = (10 ^(1/–slope)^ – 1) × 100. The relative standard curve was used to calculate the relative quantity (Rq) values of each sample for each gene (Mendes et al., 2013).

**TABLE 1:** Primers used for RT-qPCR assays.

Gene target	Forward primer (5′–3′)	Reverse primer (5′–3′)	References
*sdiA*	ACGCGCAATGTTGTTACGCT	TTCCAGCCGCTGTGTCTGAT	This study
*deoD*	TGCGCATGGACGTCAAACTG	TGCCGCATCAACTGCGTTAC	This study
*deoR*	CCTATCGGGCCAAGAGCGAT	GCCGCTGGCGGAAATAAACA	This study
*add*	ACGGCTGTAACACGTTTGGC	CCCAGCTCATCACCGGCTAA	This study
*hpt*	CCGAGCTGGGTCGTCAGATT	AACCTGGACTTCACGGCACA	This study
*16S rRNA*	CGTGTTGTGAAATGTTGGGTTAA	CCGCTGGCAACAAAGGATAA	Johny et al., 2017

### Statistical Analysis

The experiments were carried out in three biological replicates. The data of growth, pH, titratable acidity, glucose consumption, and gene expression were submitted to analysis of variance (ANOVA) and Tukey’s test using the Statistical Analysis System and Genetics Software^®^ (Ferreira, 2014). A *p* < 0.05 was considered to be statistically significant. The graphs were built on the GraphPad Prism 5.0 program (GraphPad Inc., San Diego, CA, United States). For metabolites, principal component analysis (PCA) was performed with the triplicates of each time using software R. The average values of the triplicates were used to construct the heatmap and dendrogram, using software R. The Pearson correlation analysis was also performed using software R (R Foundation for Statistical Computing, Vienna, Austria) and the metabolites with *p* < 0.05 were considered significant and used for pathway impact analysis using the Metaboanalyst 2.0 software (Xia Lab, Montreal, QC, Canada; Xia et al., 2009; Xia and Wishart, 2016), where the data of *Escherichia coli* K12 were used as a metabolic reference map. The metabolites of the pathways most impacted by the addition of C12-HSL were subjected to Tukey’s test using GraphPad Prism 5.0 program. A *p* < 0.05 was considered to be statistically significant.

## Results

### pH and Titratable Acidity

The pH and titratable acidity of the growth medium were evaluated to characterize the occurrence of sublethal acid stress and to quantify acidic compounds throughout the cultivation time. Both in the control and treatment with the addition of 50 nmol.L^–1^ C12-HSL, there was a gradual reduction of pH, and the minimum value reached 5.31 and 5.28 respectively, at 12 h incubation, and at this time the highest values of acid compounds were obtained (Supplementary Table S1). At 4, 6, and 24 h of cultivation, the culture medium with the addition of C12-HSL had slightly higher pH than the control (Supplementary Table S1). However, the difference in titratable acidity was only observed with 24 h of cultivation, and the cultivation of *Salmonella* with C12-HSL presented a higher titratable acidity (Supplementary Table S1).

### Growth and Glucose Consumption

**FIGURE 1:**
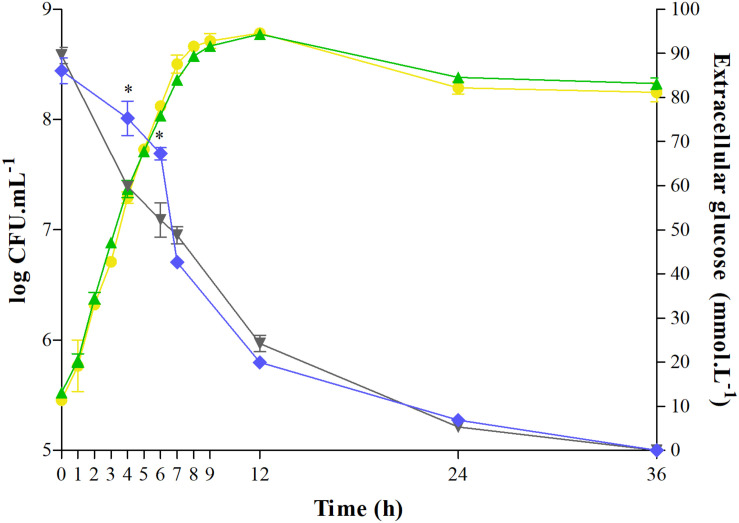
Quantification of extracellular glucose and determination of growth of *Salmonella* Enteritidis PT4 578 cultivated in anaerobic TSB at 37°C for 36 h with addition of acetonitrile (gray triangle and yellow circle, respectively) or 50 nmol L^–1^ of C12-HSL (blue diamond and green triangle, respectively). Error bars indicate a standard deviation, and * means significant difference (*p* < 0.05).

*Salmonella* growth and glucose consumption in the control or C12-HSL treatment in anaerobic TSB medium for 36 h were evaluated (Figure 1). C12-HSL has no apparent influence on the growth of *Salmonella* Enteritidis PT4 578 (Figure 1). However, the concentration of glucose in the extracellular medium with the addition of C12-HSL after 4 and 6 h of cultivation was 26 and 29% higher, respectively, indicating a lower consumption of this sugar when compared to the control (Figure 1). Despite the lower glucose consumption in the C12-HSL treatment, numbers of log CFU mL^–1^ values indicated a population similar to the control (Figure 1). On the other hand, after 7 h of cultivation, there is no difference in glucose uptake (Figure 1).

### Intracellular Metabolites

**FIGURE 2:**
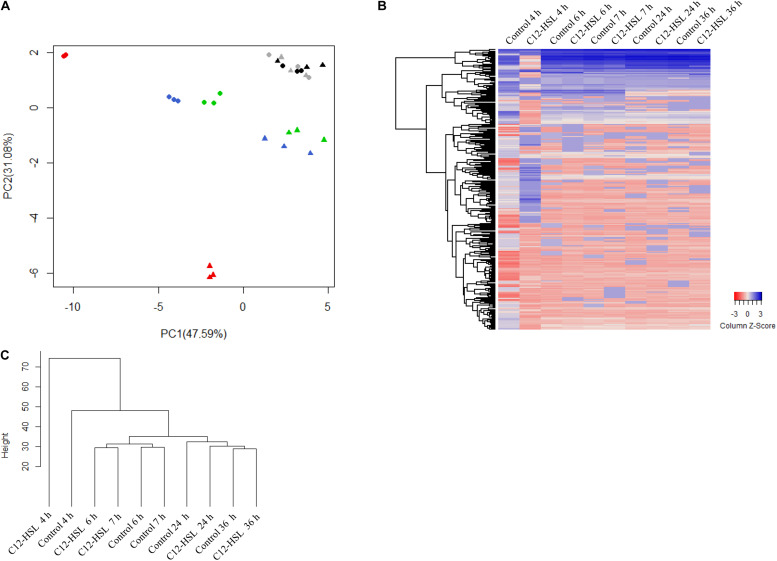
Principal component analysis (PCA) score plot of metabolites from *Salmonella* Enteritidis PT4 578 anaerobically cultivated in TSB at 37°C with addition of acetonitrile (filled triangle) or 50 nmol L^–1^ of C12-HSL (filled circle). The colors represent the times of cultivation 4 h (red), 6 h (blue), 7 h (green), 24 h (gray), and 36 h (black) **(A)**. Heatmap of the relative levels of each metabolite identified by GC-MS over time. Each row corresponds to a unique metabolite and each column at the mean of the triplicate values. The color scale ranges from red (low abundance) to blue (high abundance) **(B)**. Dendrogram of the mean values of the triplicates at each time. The height of the arms is proportional to the difference in the abundance profile of the metabolites **(C)**.

The intracellular metabolites were evaluated by GC-TOF-MS. Our results show that the addition of C12-HSL influenced the levels of a large variety of metabolites in *Salmonella.* We used principal component analysis (PCA) to evaluate the quality of replicates, to understand the data structure, and to detect data patterns. The score plot generated by submitting intracellular metabolites to PCA is demonstrated in Figure 2A. Principal component 1 (PC-1) and PC-2 explained together 88.67% of the data variance (Figure 2A). The variation between the replicates is much lower than the variation between the times, indicating that the chosen methanol concentration of 60% for the metabolic quenching was sufficient to keep the cells intact, avoiding leaks and loss of metabolites being thus, very suitable for the analysis of *Salmonella* intracellular metabolites. The PCA analysis shows that there is a clear dispersion between the C12-HSL treatment and the control in the time of 4 h and over the growing time the samples tend to cluster, demonstrating that the changes in the metabolic profile caused by C12-HSL tend to reduce over the cultivation time and, from 24 h, no difference is observed (Figure 2A).

The heatmap (Figure 2B) shows, in a global way, changes in the relative levels of each metabolite over time. The profile in the time of 4 h, in the C12-HSL treatment, has more variations, and this observation is confirmed by the dendrogram resulting from cluster analysis by agglomerative hierarchical methods (Figure 2C).

**FIGURE 3:**
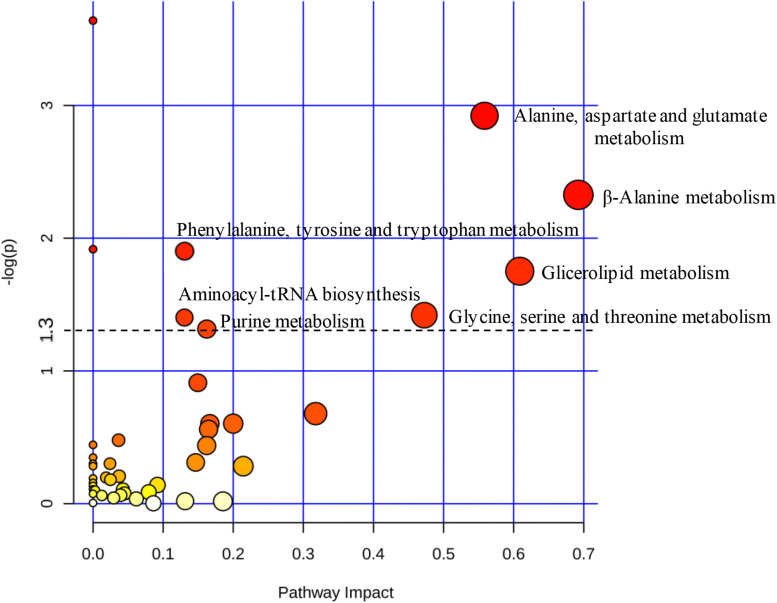
The pathway impact analysis using MetaboAnalyst 2.0. Metabolic pathways with values –log (*p*) ≥ 1.3 (*p* ≤ 0.05) were considered significant (above dashed line). The node color is based on its *p*-value and the scale of significance goes from white (highest *p*-value) to red (lowest *p*-value). The node radius is determined based on their pathway impact values.

Based on uncorrelated metabolites by Pearson’s analysis, the metabolic pathway impact revealed the distinct perturbation of C12-HSL in a total of seven pathways (Figure 3). Among these pathways, four are related to amino acid metabolism, and the others correspond to the aminoacyl-tRNA biosynthesis, metabolism of glycerolipid and purines (Figure 3 and Supplementary Table S2).

After 4 h of cultivation the levels of cyclic guanosine monophosphate (3’, 5’-Cyclic GMP), cyclic adenosine monophosphate (3’, 5’-Cyclic AMP) and the first nucleotide formed during the purine synthesis, the inosine monophosphate (IMP) decrease in comparison to the control (Supplementary Figure S1 and Supplementary Table S2). There was also a reduction in levels of nucleosides guanosine, xanthosine, and adenosine, but an increase in hypoxanthine and inosine bases levels (Supplementary Figure S1 and Supplementary Table S2). Also, there was an increase in deoxynucleoside levels, deoxyguanine, and deoxyadenosine (Supplementary Figure S1 and Supplementary Table S2).

**FIGURE 4:**
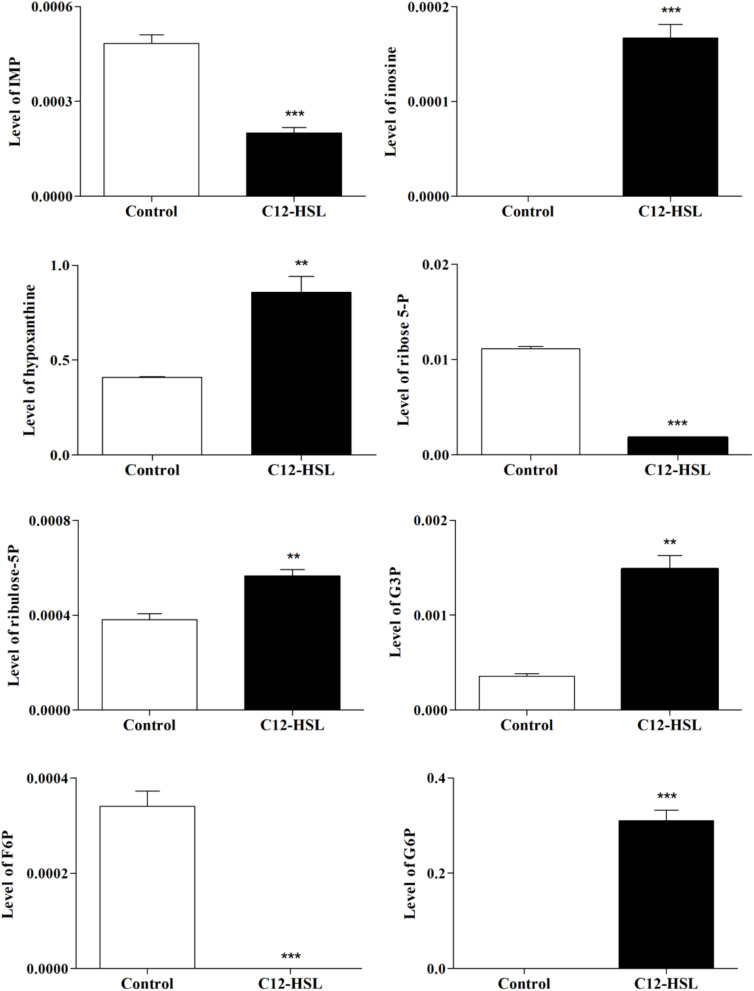
The relative levels of metabolites belonging to the glycolytic pathway and the PPP identified after 4 h of cultivation with addition of acetonitrile (white bar) or 50 nmol L^–1^ of C12-HSL treatment (black bar). Error bars indicate the standard error, and values that are significantly different to the control by Tukey’s test are indicated; **p < 0.01; ***p < 0.001. PPP, pentose phosphate pathway; IMP, inosine monophosphate; G3P, glyceraldehyde-3-phosphate; F6P, fructose-6- phosphate; G6P, glucose-6-phosphate; -P, -phosphate.

**FIGURE 5:**
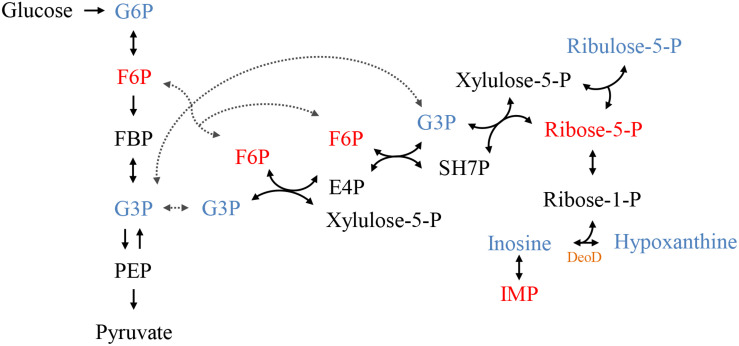
The connection between the glycolytic pathway and the pentose phosphate pathway (PPP). The colors represent the metabolites identified by GC-MS, blue represents increased levels and red, reduced levels compared to control. In black metabolites that have not been identified but complete the pathway. In orange highlighted the protein DeoD, whose gene expression was evaluated in this work. PPP, pentose phosphate pathway; G6P, glucose-6-phosphate; F6P, fructose-6-phosphate; FBP, fructose-1,6- bisphosphate; G3P, glyceraldehyde-3-phosphate; PEP, phosphoenolpyruvate; SH7P, sedoheptulose-7-phosphate; E4P, erythrose 4-phosphate; IMP, inosine monophosphate; -P, -phosphate.

The individual analysis of some identified metabolites and belonging to the glycolytic pathway and pentose phosphate pathway (PPP), showed that there is a change in their levels due to the addition of C12-HSL (Figure 4). The reduction of ribose-5-phosphate (ribose-5-P) levels may represent greater interconversion between its isomer ribulose-5-phosphate (ribulose-5-P) and increased activation of the non-oxidative branch of PPP (Figure 5).

### Expression of *sdiA* and Genes Related to the Nucleotide Salvage Pathway by RT-qPCR

**FIGURE 6:**
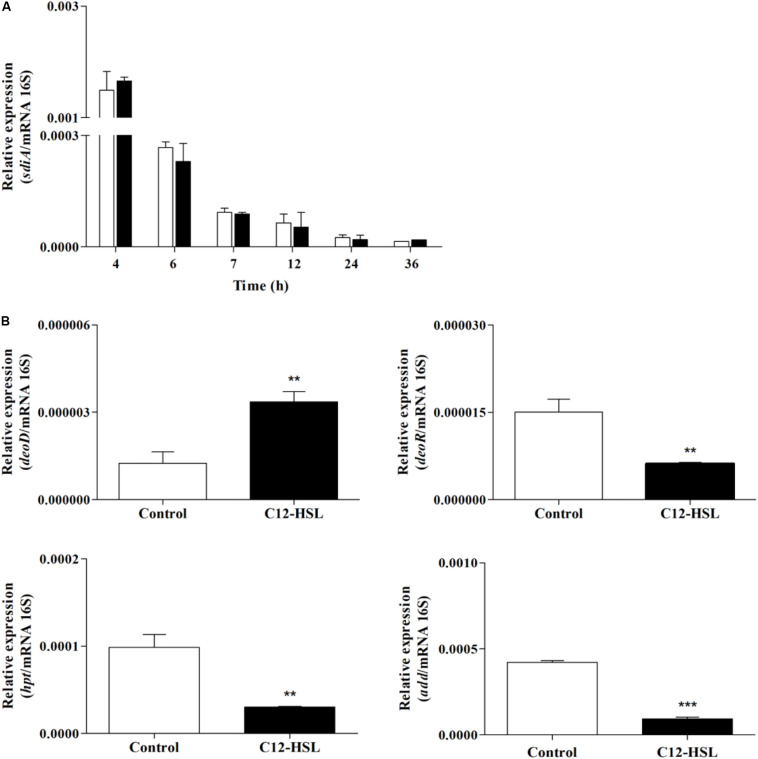
The relative gene expression of *sdiA* from *Salmonella* Enteritidis PT4 578 anaerobically cultivated in TSB at 37°C with addition of acetonitrile (white bar) or 50 nmol L^–1^ of C12-HSL (black bar) **(A)**. The relative gene expression of *deoD*, *deoR*, *add* and *hpt* from *Salmonella* Enteritidis PT4 578 after 4 h of cultivation in anaerobic TSB at 37°C in presence of acetonitrile (white bar) or 50 nmol L^–1^ of C12-HSL (black bar) **(B)**. Error bars indicate the standard deviation and values that are significantly different to the control by Tukey’s test are indicated; **p < 0.01; ***p < 0.001.

We performed an RT-qPCR analysis to evaluate *sdiA* expression and whether changes in purine metabolism were related to the expression of genes that could be related to the accumulation of hypoxanthine. For this, the expression of purine nucleoside phosphorylase (*deoD*), deoxyribose operon repressor (*deoR*), adenosine deaminase (*add*), and hypoxanthine phosphoribosyltransferase (*hpt*) genes were analyzed. The expression of *sdiA* gene was the same in the control and C12-HSL treatment in both conditions, the expression varied overtime of cultivation, being higher in the early stages (Figure 6A). Interestingly, *deoD*, which performs the reversible conversion of nucleosides to free bases and pentose-1-phosphate had the expression positively altered in the C12-HSL treatment (Figure 6B). The *deoD* gene is a distal gene of the operon *deoCABD* that is controlled by the *deoR* repressor, down-regulated in the C12-HSL treatment (Figure 6B). The *add* which encodes the protein that catalyzes the irreversible conversion of adenosine to inosine and *hpt*, which encodes the protein that catalyzes the conversion of nucleosides to nucleotides were also down-regulated (Figure 6B).

## Discussion

In this study, we characterized the metabolic response of *Salmonella* to the addition of C12-HSL under anaerobic conditions. Analysis of pH values demonstrates the occurrence of acid sublethal stress, resulting from the activation of the mixed acid fermentation pathway widely used by enterobacteria under anaerobic conditions. The growth of *Salmonella* was not altered by the addition of C12-HSL corroborating that observed by Campos-Galvão et al. (2015) and Almeida et al. (2017b) and the lower glucose consumption in the early times suggest that *Salmonella* can modulate and optimize its metabolism. For this, opting for other favorable metabolic reactions to meet carbon demands in the presence of the signaling molecule, even with glucose availability. This may be a strategy in overcrowded conditions, mimicked by the high concentration of C12-HSL. This phenotype has also been observed in other Gram-negative bacteria. Analyzes based on RNAseq of strains of the rice pathogen *Burkholderia glumae* mutated in the *N*-octanoyl-DL-homoserine lactone (C8-HSL) synthase gene (*tofI*), cognitive receptor (*tofR*), and transcriptional regulator (*qsmR*) showed significantly increased expression of *ptsI*, a representative gene in the sugar phosphotransferase (PTS) system, compared to the wild type (An et al., 2014). Moreover, by nuclear magnetic resonance spectroscopy, it was shown that the transport levels of D-glucose-1-[13C] were significantly higher in the QS mutants than in the wild type. The higher uptake of glucose by the QS mutants in *B*. *gluma*e indicates that the AI-1 QS system in this pathogen acts as a single metabolic brake when the cells are at a high population (An et al., 2014). Similar behavior was observed in the etiological agent of bubonic plague *Yersinia pestis*, in which RNAseq analyses demonstrated that the *ptsI* gene was downregulated in the wild-type compared to an AHL-null strain during the logarithmic growth phase (LaRock et al., 2013). The equal glucose absorption levels after 7 h of cultivation (Figure 1) can be justified by a 1.57-fold increase in the abundance of PtsI protein (phosphoenolpyruvate protein phosphotransferase) in *Salmonella* Enteritidis PT4 578 at the same time and cultivation in TSB anaerobic medium supplemented with C12-HSL reported by Almeida et al. (2017a).

Interestingly, the addition of C12-HSL resulted in changes in the metabolic profile during the early stages of growth, 4, 6, and 7 h and after these times, the metabolic profile was not significantly altered (Figure 2). This may be related to *sdiA* expression, which is higher in the early times (Figure 6A). Just like *Salmonella*, in enterohemorrhagic *E. coli* (EHEC) AHLs regulate SdiA at the post-transcriptional level, and the expression of *sdiA* is not affected by the signal molecule (Nguyen et al., 2015). The growth phase, as well as the accumulation of signaling molecules in the medium, were also determinant for the regulation of population density-dependent genes in *Burkholderia thailandensis* and *Pseudomonas aeruginosa* (Schuster et al., 2003; Wagner et al., 2003; Majerczyk et al., 2014).

According to the results presented in the PCA and the dendrogram, 4 h is the time that presents the biggest differences between control and C12-HSL treatment, highlighting the pathway of purine metabolism. After 4 h of cultivation, there was also the difference between glucose absorption. Since just *deoD* had its expression increased after 4 h, we hypothesized that instead of using bases to form nucleotides, in the presence of AHL, *Salmonella* can break nucleosides to release ribose-1-phosphate that could enter the PPP, generating fructose-6-phosphate and glyceraldehyde-3-phosphate that can follow glycolytic pathway or be converted to hexose and channeled into glycolysis (Figure 5). This reaction could be an interesting alternative both for obtaining energy and for producing glucose, which can be used to form other important cellular structures, thus support the growth with lower consumption of glucose.

Proteomic analysis of the adaptive response to anaerobic conditions of *Salmonella* Typhimurium cultured in nutrient broth demonstrated that the level of protein DeoD and two enzymes transketolase (TktA) and transaldolase (TalB), which are part of the non-oxidative reactions of the PPP, were induced anaerobically (Encheva et al., 2009). Moreover, the TalB protein had its abundance increased in the presence of C12-HSL after 7 h of cultivation in anaerobiosis (Almeida et al., 2017a). Another protein that has drastically increased its expression when *Salmonella* has grown in Luria-Bertani (LB) broth under anaerobic conditions is Tsx, which encodes a porin that facilitates the import of nucleosides (Bucarey et al., 2005). The expression of the *tsx* gene is controlled by two differentially regulated promoters; one of these promoters is down-regulated by the DeoR repressor (Bremer et al., 1988; Bucarey et al., 2006). Based on these data, we can infer that with the addition of C12-HSL and consequent negative regulation of the *deoR*, can alleviate the repression of the promoter that regulates the expression of Tsx, increasing the synthesis of porins and consequently, the increase of nucleoside internalization.

In *P. aeruginosa*, AHL signals positively regulate a nucleoside hydrolase (Nuh) which does the hydrolytic degradation of purine nucleotides to nucleobase and ribose (Heurlier et al., 2005). The significance of the QS control of Nuh is not fully understood, but it is added that it is a way of using the products generated during the synthesis of methylthioadenosine, a byproduct of AHL biosynthesis (Heurlier et al., 2006).

Although the significance of QS control in purine metabolism of *Salmonella* is not fully understood, the variation in expression and accumulation of metabolites provides a clear-cut metabolic phenotype that is under QS control, and this work is the first to provide evidence that the reason is for optimization of metabolism.

## Conclusion

Although some studies have already investigated the mechanism of QS in *Salmonella*, this is the first study that focused quantitatively on the response of *Salmonella* Enteritidis to AI-1 under anaerobic conditions. The results show that the addition of C12-HSL changes the metabolic profile and reduces glucose absorption by *Salmonella* Enteritidis under anaerobic conditions. However, despite the reduction of glucose consumption, the growth is not affected, suggesting that *Salmonella* can use alternative pathways that make pentose moiety of exogenous nucleosides available as sources of carbon and optimize its metabolism in conditions of high population density. The changes in the metabolic profile accentuated in the initial stages of growth corroborate with the literature data and may be related to the highest expression of the response protein, SdiA. Given the limited number of studies on the processes regulated by QS, specifically AI-1, in *Salmonella*, this work brings new information on the influence of this signaling molecule on the metabolism of the pathogen most frequently reported in outbreaks involving the consumption of contaminated foods. Besides, it reinforces the role of QS in the synchronization of microbial metabolism to changes that can occur in a super crowded environment.

## Data Availability Statement

All datasets generated for this study are included in the article/Supplementary Material.

## Author Contributions

DC, FA, TM, and MV: conceptualization and experiment design. DC, FA, TM, AA, and NV: data curation. MV and TM: funding acquisition. DC, FA, AA, and NV: performing the experiments. MV: project administration and supervision. DC: validation. DC, FA, TM, UP, and MV: writing original draft. All authors contributed to the article and approved the submitted version.

## Conflict of Interest

The authors declare that the research was conducted in the absence of any commercial or financial relationships that could be construed as a potential conflict of interest.

## Footnotes

1 http://gmd.mpimp-golm.mpg.de/

2 https://www.metabolomicsworkbench.org
