# Supplementary material for: Salmonella enterica Optimizes Metabolism After Addition of Acyl-Homoserine Lactone Under Anaerobic Conditions
Source: Front Microbiol. 2020 Jul 28;11:1459. doi: 10.3389/fmicb.2020.01459 (PMC7401450; doi:10.3389/fmicb.2020.01459)
Supplement: Supplementary file 1 [file Table_1.docx]

***Supplementary Material***

**Supplementary Table S1.** Evaluation of the pH and titratable acidity of the medium throughout the cultivation time of *Salmonella* Enteritidis PT4 578 in anaerobic TSB with or without the addition of 50 nmol L^-1^ of C12-HSL.

| **Time (h)** | **pH** | |  | **Titratable acidity (eq.L^-1^)** | |
| --- | --- | --- | --- | --- | --- |
|  | Control | C12-HSL |  | Control | C12-HSL |
| **0** | 6.45^A^ | 6.45^A^ |  | 16.06^E^ | 16.06^E^ |
| **4** | 5.81^bC^ | 5.90^aB^ |  | 26.10^D^ | 26.10^D^ |
| **6** | 5.49^bE^ | 5.56^aE^ |  | 30.12^C^ | 30.12^C^ |
| **7** | 5.35^F^ | 5.38^F^ |  | 32.13^B^ | 32.13^B^ |
| **12** | 5.31^F^ | 5.28^G^ |  | 34.14^A^ | 34.14^A^ |
| **24** | 5.54^bD^ | 5.61^aD^ |  | 30.12^bC^ | 32.13^aB^ |
| **36** | 5.86^B^ | 5.83^C^ |  | 30.79^C^ | 30.13^B^ |

The evaluation was made between control and C12-HSL treatment at specific times and over time. Different lowercase letters on the same line (between control and C12-HSL treatment) and different capital letters in the same column (over time) differs at 5% probability (*p* < 0.05) by Tukey's test. When no lower case letter is shown, there is no statistical difference between control and C12-HSL treatment.
