# Supplementary material for: Salmonella enterica Optimizes Metabolism After Addition of Acyl-Homoserine Lactone Under Anaerobic Conditions
Source: Front Microbiol. 2020 Jul 28;11:1459. doi: 10.3389/fmicb.2020.01459 (PMC7401450; doi:10.3389/fmicb.2020.01459)
Supplement: Supplementary file 2 [file Table_2.docx]

***Supplementary Material***

**Supplementary Table S2.** Variation of the metabolites of the pathways most impacted by the addition of 50 nmol L^-1^ of C12-HSL in relation to the control.

| Time (h) | 4 | | 6 | | 7 | | | | 24 | | | | 36 | | | | |
| --- | --- | --- | --- | --- | --- | --- | --- | --- | --- | --- | --- | --- | --- | --- | --- | --- | --- |
| Pathaway / Metabolite | log_2_ ^T/C^ | *p* value | log_2_ ^T/C^ | *p* value | log_2_ ^T/C^ | | *p* value | | log_2_ ^T/C^ | | *p* value | | log_2_ ^T/C^ | | *p* value | | |
| Alanine, aspartate and glutamate metabolism | | | | | | | | | | | | | | | | | |
| L-Aspartate | -12.90 | < 0.0001 | -0.93 | 0.0003 | -0.92 | | 0.0038 | | -0.24 | | 0.3273 | | -0.13 | | 0.0933 | | |
| L-Glutamine | -7.39 | 0.0003 | 0.32 | 0.1328 | >0.00 | | < 0.0001 | | - | | - | | <0.00 | | 0.0001 | | |
| L-Asparagine | -2.41 | < 0.0001 | -1.40 | 0.0004 | -1.15 | | 0.0022 | | 0.76 | | 0.0203 | | -0.14 | | 0.2264 | | |
| Oxaloacetate | -7.77 | < 0.0001 | -1.12 | 0.1481 | 1.2 | | 0.1181 | | 0.53 | | 0.0788 | | 1.51 | | 0.003 | | |
| L-Carbamoyl-L-Aspartate | -4.13 | < 0.0001 | -0.94 | < 0.0001 | -0.42 | | 0.2235 | | >0.00 | | 0.0008 | | -1.02 | | 0.0026 | | |
| 4-Aminobutanoato | -11.39 | 0.0002 | - | - | - | | - | | - | | - | | >0.00 | | < 0.0001 | | |
| Beta Alanine metabolism | | | | | | | | | | | | | | | | | |
| L-Aspartate | -12.90 | < 0.0001 | -0.93 | 0.0003 | -0.92 | | 0.0038 | | -0.24 | | 0.3273 | | -0.13 | | 0.0933 | | |
| Spermine | -2.64 | 0.0023 | -0.09 | 0.3527 | -0.44 | | 0.0102 | | 0.00 | | < 0.0001 | | 0.19 | | 0.0296 | | |
| Beta-Alanine | >0.00 | 0.0006 | 0.15 | 0.1814 | -1.10 | | 0.0115 | | 0.14 | | 0.4499 | | -0.69 | | 0.0047 | | |
| L-Histidine | -7.90 | 0.0001 | 0.18 | 0.3246 | -0.38 | | 0.0301 | | -0.02 | | 0.956 | | 0.43 | | 0.0986 | | |
| 4-Aminobutanoato | -11.39 | 0.0002 | - | - | - | | - | | - | | - | | >0.00 | | < 0.0001 | | |
| Glycine, serine and threonine metabolism | | | | | | | | | | | | | | | | | |
| L-Aspartate | -12.90 | < 0.0001 | -0.93 | 0.0003 | -0.92 | | 0.0038 | | -0.24 | | 0.3273 | | -0.13 | | 0.0933 | | |
| L-Threonine | >0.00 | 0.0004 | -1.07 | 0.0024 | -1.24 | | 0.0006 | | 0.05 | | 0.7095 | | -0.06 | | 0.4929 | | |
| L-Glycine | -12.40 | < 0.0001 | -1.27 | 0.0011 | -0.20 | | 0.4183 | | -0.21 | | 0.1603 | | -0.13 | | 0.4009 | | |
| Glycerate | >0.00 | 0.0005 | -1.43 | 0.0055 | -1.70 | | 0.0011 | | 0.59 | | 0.0234 | | 0.21 | | 0.1765 | | |
| Hydroxypyruvate | -0.13 | 0.1786 | 0.01 | 0.9695 | -1.14 | | < 0.0001 | | 0.42 | | 0.0037 | | 0.60 | | < 0.0001 | | |
| L-Serine | -13.02 | < 0.0001 | -0.66 | 0.0068 | -1.38 | | 0.0039 | | 0.58 | | 0.0033 | | 0.16 | | 0.3321 | | |
| L-Tryptophan | -4.00 | < 0.0001 | -0.77 | < 0.0001 | -0.30 | | 0.1606 | | -0.39 | | 0.0251 | | 0.39 | | 0.1289 | | |
| Phenylalanine, tyrosine and tryptophan biosynthesis | | | | | | | | | | | | | | | | | |
| Phosphoenolpyruvate | - | - | 0.83 | 0.0017 | - | | - | | <0.00 | | < 0.0001 | | - | | - | | |
| 2-Dehydro-3-deoxy-D-arabino-heptonate 7-phosphate | -6.02 | < 0.0001 | -0.19 | 0.5491 | - | | - | | <0.00 | | < 0.0001 | | 0.47 | | 0.021 | | |
| Shikimate 3-phosphate | - | - | - | - | >0.00 | | < 0.0001 | | 0.12 | | 0.4054 | | >0.00 | | 0.0056 | | |
| Phenylpyruvate | <0.00 | 0.0005 | >0.00 | < 0.0001 | -0.60 | | 0.1409 | | - | | - | | <0.00 | | < 0.0001 | | |
| L-Phenylalanine | -4.49 | < 0.0001 | -0.95 | 0.001 | -0.85 | | 0.0002 | | 0.08 | | 0.7077 | | 0.02 | | 0.8465 | | |
| L-Tryptophan | -4.00 | < 0.0001 | -0.77 | < 0.0001 | -0.30 | | 0.1606 | | -0.39 | | 0.0251 | | 0.38 | | 0.1289 | | |
| Aminoacyl-tRNA biosynthesis | | | | | | | | | | | | | | | | |  |
| L-Asparagine | -2.41 | < 0.0001 | -1.40 | 0.0004 | -1.15 | 0.0022 | | | 0.76 | | 0.0203 | | -0.14 | | 0.2264 | |  |
| L-Histidine | -7.90 | 0.0001 | 0.18 | 0.3246 | -0.39 | 0.0301 | | | -0.02 | | 0.956 | | 0.43 | | 0.0986 | |  |
| L-Phenylalanine | -4.49 | < 0.0001 | -0.95 | 0.001 | -0.85 | 0.0002 | | | 0.08 | | 0.7077 | | 0.02 | | 0.8465 | |  |
| L-Glutamine | -7.39 | 0.0003 | 0.32 | 0.1328 | >0.00 | < 0.0001 | | | - | | - | | <0.00 | | 0.0001 | |  |
| L-Glycine | -12.40 | < 0.0001 | -1.27 | 0.0011 | -0.19 | 0.4183 | | | -0.21 | | 0.1603 | | -0.13 | | 0.4009 | |  |
| L-Aspartate | -12.90 | < 0.0001 | -0.93 | 0.0003 | -0.92 | 0.0038 | | | -0.24 | | 0.3273 | | -0.13 | | 0.0933 | |  |
| L-Serine | -13.02 | < 0.0001 | -0.66 | 0.0068 | -1.38 | 0.0039 | | | 0.58 | | 0.0033 | | 0.16 | | 0.3321 | |  |
| L-Valine | -10.79 | < 0.0001 | -1.16 | 0.0108 | -1.27 | 0.0003 | | | 0.02 | | 0.9197 | | -0.06 | | 0.493 | |  |
| L-Lysine | -9.77 | < 0.0001 | -0.67 | 0.0357 | -0.97 | 0.0016 | | | -0.03 | | 0.7236 | | -0.01 | | 0.809 | |  |
| L-Isoleucine | -14.23 | < 0.0001 | -1.12 | 0.0057 | -1.19 | 0.0006 | | | 0.10 | | 0.6135 | | -0,07 | | 0.4518 | |  |
| L-Leucine | -13.14 | < 0.0001 | -0.96 | 0.0014 | -0.92 | 0.0037 | | | -0.16 | | 0.3656 | | -0.17 | | 0.4454 | |  |
| L-Threonine | >0.00 | 0.0004 | -1.70 | 0.0024 | -1.24 | 0.0006 | | | 0.05 | | 0.7095 | | -0.06 | | 0.4929 | |  |
| L-Tryptophan | -4.00 | < 0.0001 | -0.77 | < 0.0001 | -0.30 | 0.1606 | | | -0.39 | | 0.0251 | | 0.39 | | 0.1289 | |  |
| Purine metabolism | | | | | | | | | | | | | | | | |  |
| Sulfate | -1.59 | < 0.0001 | -0.32 | 0.1205 | -1.05 | 0.0049 | | | 0.09 | | 0.5141 | | 0.01 | | 0.8413 | |  |
| L-Glutamine | -7.39 | 0.0003 | 0.32 | 0.1328 | >0.00 | < 0.0001 | | | - | | - | | <0.00 | | 0.0001 | |  |
| 3',5'-cyclic AMP | -5.44 | 0.0002 | -0.42 | 0.1582 | - | - | | | >0.00 | | 0.001 | | <0.00 | | 0.0004 | |  |
| 3',5'-Cyclic GMP | <0.00 | < 0.0001 | -0.36 | 0.0037 | -0.77 | 0.0086 | | | <0.00 | | 0.0025 | | - | | - | |  |
| Deoxyguanosine | >0.00 | 0.0007 | <0.00 | < 0.0001 | -0.66 | 0.0095 | | | <0.00 | | 0.0002 | | >0.00 | | 0.0006 | |  |
| Guanine | - | - | -0.27 | 0.1291 | <0.00 | 0.0081 | | | <0.00 | | < 0.0001 | | <0.00 | | < 0.0001 | |  |
| Inosinic acid | -1.30 | 0.0009 | -0.52 | 0.0203 | <0.00 | < 0.0001 | | | -0.35 | | 0.0706 | | 0.35 | | 0.024 | |  |
| Guanosine | -7.02 | < 0.0001 | <0.00 | < 0.0001 | 0.07 | 0.6992 | | | >0.00 | | < 0.0001 | | >0.00 | | < 0.0001 | |  |
| Adenosine | <0.00 | < 0.0001 | 0.34 | 0.0615 | <0.00 | 0.0018 | | | 0.05 | | 0.7905 | | >0.00 | | < 0.0001 | |  |
| Adenine | -11.35 | 0.0001 | -0.87 | 0.0097 | -0.77 | 0.0024 | | | 0.15 | | 0.3968 | | -0.07 | | 0.7115 | |  |
| Inosine | >0.00 | 0.0005 | <0.00 | 0.0013 | -0.31 | 0.2823 | | | >0.00 | | < 0.0001 | | -0.60 | | 0.0749 | |  |
| Deoxyadenosine | >0.00 | 0.0062 | -0.39 | 0.0083 | >0.00 | < 0.0001 | | | -0.56 | | 0.3428 | | 0.18 | | 0.4183 | |  |
| Hypoxanthine | 1.07 | 0.0059 | -1.18 | 0.0002 | -0.92 | 0.0017 | | | 0.09 | | 0.9451 | | 0.09 | | 0.4475 | |  |
| Xanthosine | <0.00 | 0.0009 | >0.00 | < 0.0001 | -0.23 | 0.2466 | | | 0.06 | | 0.7056 | | >0.00 | | < 0.0001 | |  |
| Glycerolipid metabolism | | | | | | | | | | | | | | | |  |  |
| Glycerol | >0.00 | 0.0012 | -1.29 | 0.0004 | -1.32 | 0.0013 | | -0.13 | | 0.3174 | | -0.04 | | 0.7049 | |  |  |
| Glycerate | >0.00 | 0.0005 | -1.43 | 0.0055 | -1.70 | 0.0011 | | 0.60 | | 0.0234 | | 0.21 | | 0.1765 | |  |  |
| *sn*-Glycerol-3-Phosphate | -6.55 | < 0.0001 | -1.15 | 0.0012 | -0.95 | 0.0042 | | 0.05 | | 0.68 | | 0.21 | | 0.3784 | |  |  |
| Glycerone | -1.20 | 0.001 | -0.05 | 0.847 | 0.16 | 0.283 | | >0.00 | | < 0.0001 | | -0.29 | | 0.2382 | |  |  |

Log_2_ of the treatment (T)/control (C) ratio= log_2_ ^T/C^; Metabolite not detected in the control = >0.00; Metabolite not detected in the treatment = <0.00.
