# Supplementary material for: Salmonella enterica Optimizes Metabolism After Addition of Acyl-Homoserine Lactone Under Anaerobic Conditions
Source: Front Microbiol. 2020 Jul 28;11:1459. doi: 10.3389/fmicb.2020.01459 (PMC7401450; doi:10.3389/fmicb.2020.01459)
Supplement: Supplementary file 3 [file Image_1.pdf]

Supplementary Material

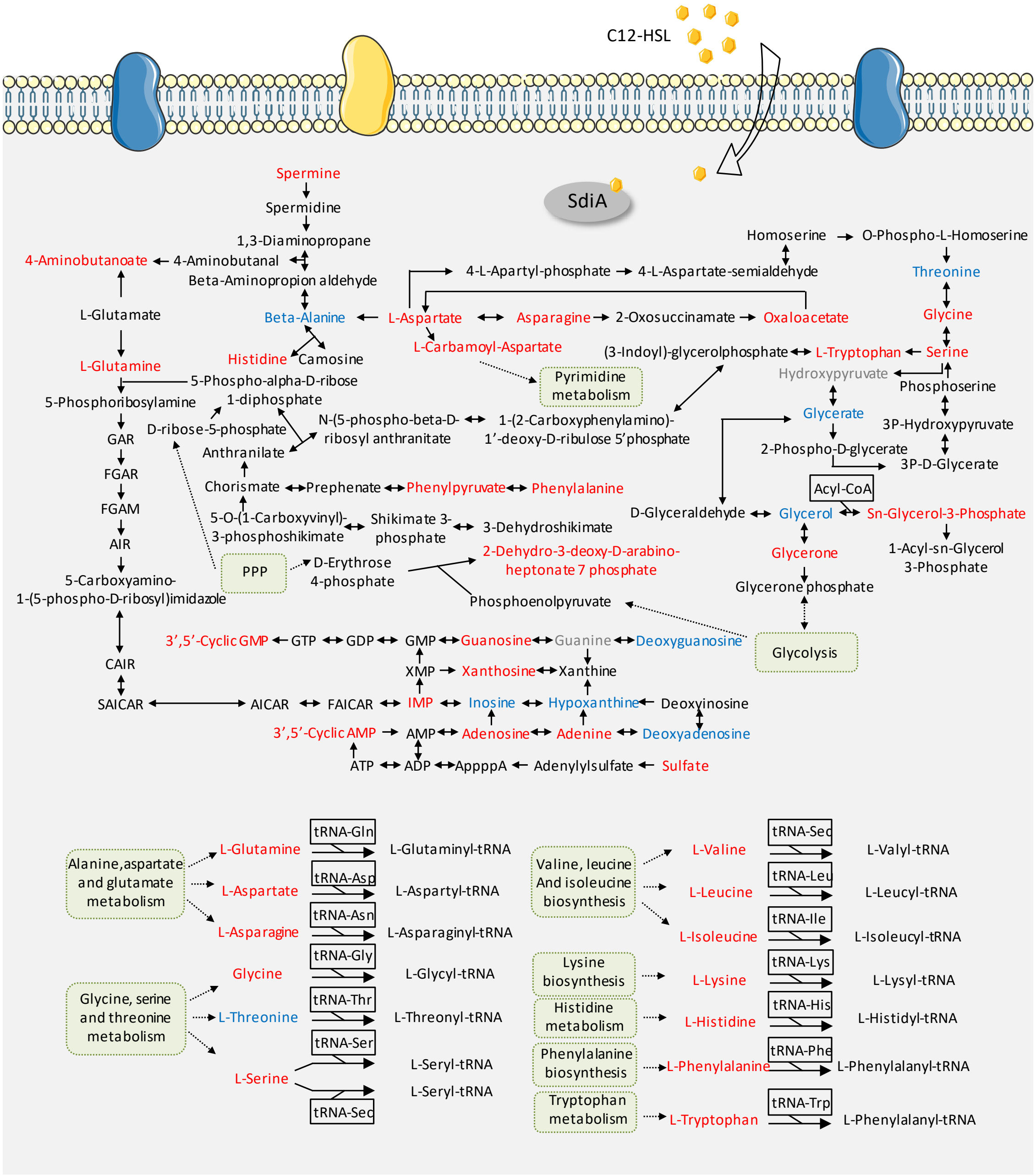

Supplementary Figure S1. Pathways altered in the C12-HSL treatment in relation to the control after 4 h of cultivation. The metabolites that had their levels increased in the C12-HSL treatment are shown in blue, those that have been reduced are shown in red, those that did not differ in gray and those that have not been identified but complete the pathway in black. Abbreviations: GAR, 5'-Phosphoribosylglycinamide; FGAR, 5'-Phosphoribosyl-N-formylglycinamide; FGAM, 2-(Formamido)-N1-(5'-phosphoribosyl)acetamidine; AIR, Aminoimidazole ribotide; CAIR, 1-(5-Phospho-D-ribosyl)-5-amino-4-imidazolecarboxylate; SAICAR, 1-(5'-Phosphoribosyl)-5-amino-4-(N-succinocarboxamide)-imidazole; AICAR, 1-(5'-Phosphoribosyl)-5-amino-4-imidazolecarboxamide; FAICAR, 1-(5'-Phosphoribosyl)-5-formamido-4-imidazolecarboxamide; IMP, Inosine monophosphate; AMP, Adenosine 5'-monophosphate; ADP, Adenosine 5'-diphosphate; ATP, Adenosine 5'-triphosphate; XMP, Xanthosine 5'-phosphate; GMP, Xanthosine 5'-phosphate; GDP, Guanosine 5'-diphosphate; GTP, Guanosine 5'-triphosphate; ApppA, Adenosine 5'-triphosphate 5'-adenosine.
